# Repression of metadherin inhibits biological behavior of prostate cancer cells and enhances their sensitivity to cisplatin

**DOI:** 10.3892/mmr.2015.3357

**Published:** 2015-02-16

**Authors:** YONG-BAO WEI, QIONG GUO, YUN-LIANG GAO, BIN YAN, ZHAO WANG, JIN-RUI YANG, WEI LIU

**Affiliations:** 1 Department of Urology, The Second Xiangya Hospital, Central South University, Changsha, Hunan 410011, P.R. China; 2 Department of Urology, Fujian Provincial Hospital, The Teaching Hospital of Fujian Medical University, Fuzhou 350001, P.R. China; 3 Department of Urology, Changsha Central Hospital, Changsha, Hunan 410004, P.R. China; 4 Department of Respiratory Medicine, The Third Hospital of Changsha, Changsha, Hunan 410015, P.R. China

**Keywords:** metadherin, astrocyte-elevated gene-1, prostate cancer, chemotherapy, cisplatin, phosphoinositide 3-kinase/Akt

## Abstract

Metadherin *(MTDH)*, also known as astrocyte-elevated gene-1, was first cloned in 2002 and has been confirmed as an oncogene in numerous types of cancer by previous studies. Overexpression of *MTDH* has been observed in multiple types of cancer, including breast, esophageal, prostate, cervical and non-small-cell lung cancer, as well as neuroblastoma and hepatocellular carcinoma. However, at present, few investigations into *MTDH*-associated prostate cancer have been performed. A previous study suggested that *MTDH* was expressed at higher levels in prostate cancer samples, compared with those of benign prostatic hyperplasia. The present study aimed to elucidate the effects of *MTDH* as an oncogene associated with the biological behavior of prostate cancer cells and chemotherapy-sensitivity to cisplatin *in vitro*. It was demonstrated that the inhibition of *MTDH* expression promoted cell apoptosis, reduced cell viability and weakened the invasive ability of prostate cancer cells. In addition, the suppression of *MTDH* expression increased cell sensitivity to cisplatin. Furthermore, it was demonstrated that *MTDH*-associated phosphoinositide 3-kinase/Akt signaling pathways may be involved in mediating the biological behavior of prostate cancer.

## Introduction

Prostate cancer (PC) is one of the most common types of cancer amongst males worldwide (1). In the United States of America, PC is the most common form of male malignancy. The occurrence and development of tumors are associated with gene mutation and disorders of signal transduction pathways; therefore, treatments aimed at targeting these abnormal genes and pathways may provide a novel focus for the development of cancer therapeutics (2). Patients with PC may also benefit from the development of such therapeutics. Recently, PC suppressor genes and oncogenes have been identified and have emerged as a significant area of study among researchers.

Metadherin (*MTDH*) is also known as astrocyte-elevated gene-1 (3,4), and was first cloned in 2002 (4). *MTDH* has been confirmed as an oncogene by multiple studies (4–12). Results taken from *in vitro* data and findings from the analysis of tissue specimens have confirmed that *MTDH* expression is significantly higher in cancerous tissue than in peritumoral tissue or normal cells, this comparison includes hepatocellular carcinoma (5,6), malignant glioma (7), breast cancer (8), renal cell carcinoma (9), neuroblastoma cell lines (10) and PC (11–13). *MTDH* is not only overexpressed in numerous types of cancer, but is also involved in tumor metastasis. Since 2004, *MTDH* has been considered a potential mediator of cancer metastasis involving lung metastases from breast cancer (3). *In vivo* and *in vitro*, it has been demonstrated that the 8q22 genomic gain increases expression of the dual-function metastasis gene *MTDH* (14). In addition, a further study indicated that *MTDH* promotes angiogenesis (15). *MTDH* overexpression enhances human umbilical vein endothelial cell formation, while *MTDH* knockout has opposing effects (15). These previous studies have confirmed that *MTDH* may activate signaling transduction pathways associated with tumor development, which may influence the biological features of the tumors. These features are characterized as transformation, tumor escape, apoptosis, proliferation, invasion, metastasis, angiogenesis and chemotherapy resistance (5–15).

To the best of our knowledge, to date, only few studies have been conducted investigating the association between *MTDH* and PC. However, there is evidence demonstrating that *MTDH* is expressed at higher levels in PC samples, compared with those of benign prostatic hyperplasia (12). Previous *in vitro* studies have revealed that *MTDH* regulates FOXO3a protein activity (11) and BCCIPα expression (13) using PC cells.

Cisplatin is a platinum compound that has been available since 1978, and is currently recommended for the treatment of few types of cancer (16), including PC. A previous study demonstrated the addition of a low dose of cisplatin enhanced the effects of a standard dose of 89Sr, without significant side effects, and produced a significant improvement in pain palliation and a cytostatic effect on bone disease from PC (17). Recently, targeted delivery of cisplatin has been shown to markedly improve its tolerability and efficacy in prostate cancer therapy *in vivo* (18). The present study aimed to elucidate the effects of *MTDH* as an oncogene in the biological behavior of PC and chemotherapy sensitivity to cisplatin *in vitro*.

## Materials and methods

### Cell culture and transfection

The human PC cell lines PC3, DU145 and LNCap were obtained from the Cell Bank of the Chinese Academy of Sciences (Beijing, China). Cells were cultured in RPMI-1640 (Gibco Life Technologies, Carlsbad, CA, USA), in a humidified incubator at 37°C containing 5% CO_2_. Three small interfering RNAs (siRNAs) for *MTDH* intevention were all purchased from Shanghai GenePharma Technology Co., Ltd. (Shanghai, China). Their sequences are as follows: *MTDH*-744 sense, 5′-GCUGUUCGAACACCUCAAATT-3′, antisense, 5′-UUUGAGGUGUUCGAACAGCTT-3′; *MTDH*-1432 sense, 5′-GCCGUAAUCAACCCUAUAUTT-3′, antisense, 5′-AUAUAGGGUUGAUUACGGCTT-3′; and *MTDH*-1883 sense, 5′-GCCAUCUGUAAUCUUAUCATT-3′, and antisense, 5′-UGAUAAGAUUACAGAUGGCTT-3′. The LNCap cells were divided into five groups: Two control groups of conventional cultured LNCap cells and LNCap cells transfected with an empty vector (Shanghai GenePharma Technology Co., Ltd.), and three interventional groups of LNCap cells transfected with *MTDH*-744, *MTDH*-1432 and *MTDH*-1883. *MTDH* intervention sequences were transfected at working concentrations, according to manufacturer’s insructions, using Lipofectamine^®^ 2000 reagent (Invitrogen Life Technologies, Carlsbad, CA, USA). Briefly, 250 *μ*l Opti-MEM^®^ I (Invitrogen Life Technologies) was added to dilute siRNA (2 *μ*M) and Lipofectamine^®^ 2000 (0.02 mg/ml), respectively. After 5 min, the two dilutions were mixed together, in order to prepare the siRNA-Lipofectamine^®^ 2000 complex. The cells (5×10^5^ per well in six-well plates) were then transfected with the siRNA-Lipofectamine^®^ 2000 complex and cultured for 48h at 37°C, in an atmosphere containing 5% CO_2_. The transfection efficiency was then assessed by measuring the percentage of transfected cells via microscopy, and *MTDH* protein expression levels were determined in each group; *MTDH*-1432 was selected for further studies. Untransfected cells were the control cells.

### Optimum concentration of cisplatin

Based on the levels of *MTDH* expression, the LNCap cell line was selected for use in the present experiment. Cisplatin was purchased from a subsidiary of Selleck Chemicals (Houston, TX, USA); Shanghai Blue Wood Chemical Co. (Shanghai, China). Various concentrations of cisplatin (0, 0.1, 0.5, 1.0, 5.0, 10.0, 20.0 and 50.0 *μ*g/ml) were selected and added to the culture medium, ensuring that the cell viability of the LNCap cell culture remained at ~80% following 24 h of treatment. An MTT assay (Sigma-Aldrich, St. Louis, MO, USA) was conducted to assess cell viability. A curve was constructed to select the optimum concentration of cisplatin, with cell viability and cisplatin concentration on the y- and x-axes, respectively.

### Experimental groups

The experimental groups were designated as follows: Control group A, untreated LNCap cells; intervention group B, LNCap + *MTDH* intervention sequence; control group C, LNCap + cisplatin and intervention group D, LNCap + *MTDH* intervention sequence + cisplatin. All cells were harvested following 24 h (37°C) of treatment with cisplatin and/or the *MTDH* intervention sequence.

### MTT assay

Cells were plated in 96-well plates at 1×10^4^ cells/well in a final volume of 100 *μ*l, and treated with *MTDH* intervention sequences and/or cisplatin. MTT was added following incubation for 24 h in a humidified incubator at 37°C with 5% CO_2_. Dilution buffer (25 ml; Sigma-Aldrich) was subsequently added and the plates were incubated for a further 4 h. Following removal of the culture medium, dimethyl sulfoxide (Sigma-Aldrich) was administered to the cells at 37°C for 10 min. The absorbance was measured at 570 nm using a microplate reader (SpectraMax^®^ 340PC384; Molecular Devices, Sunnyvale, CA, USA).

### Apoptosis assay

Cell apoptosis was detected using an Annexin V-fluorescein isothiocyanate (FITC)-labeling kit purchased from Nanjing Kaiji Biotech Company (Nanjing, China) and was performed according to the manufacturer’s instructions. FITC-labeled cells were counted and analyzed using the FACS Aria™ flow cytometer (BD Biosciences, Franklin Lakes, NJ, USA)

### Transwell chamber invasion assay

Matrigel (BD Biosciences) was used according to the manufacturer’s instructions. Following dilution with fetal bovine serum (FBS)-free RPMI-1640 (Sigma-Aldrich) at a ratio of 1:8, the Matrigel was added to the bottom chamber of the Transwell. LNCap cells in the exponential growth stage were treated with 0.25% tryptase and added to RPMI-1640 to produce a 1×10^6^/ml single-cell suspension. A Transwell chamber was placed into a 24-well plate. A total of 600 *μ*l of RPMI-1640 containing 10% FBS (Gibco Life Technologies) and 200 *μ*l of the prepared single-cell suspension were added. The cells were cultured at 37°C with 5% CO_2_ for 24 h. Subsequently, the liquid was removed from the Transwell chamber and the bottom chamber. The membrane was then washed three times with phosphate buffered saline, immersed in methanol (Sigma-Aldrich) and maintained for 20 min at room temperature, followed by hematoxylin staining for 10 min. The cells that had migrated through the pores to the lower surface of the membrane were counted under a microscope (magnification, ×400; TS100; Nikon, Tokyo, Japan).

### Western blot analysis

Protein lysates were separated using a 10% SDS-PAGE (Sigma-Aldrich) and transferred onto nitrocellulose blotting membranes (Pierce Biotechnology, Inc., Rockford, IL, USA). The blots were incubated with rabbit monoclonal immunoglobulin G (IgG) *MTDH* antibody which was purchased from Abcam (1:1,000; cat. no. ab124789; Abcam, Cambridge, MA, USA). The membranes were visualized using horseradish peroxidase-conjugated goat anti-rabbit IgG (1:40,000; cat. no. 14-13-06; KPL, Inc., Gaithersburg, MD, USA). GAPDH was used as a control.

### Reverse transcription quantitative polymerase chain reaction (RT-qPCR)

Total RNA was extracted from the cells using a TRIzol RNA extraction kit (Qiagen, Valencia, CA, USA). RT-qPCR was performed using an All-in-One™ qPCR mix (GeneCopoeia, Rockville, MD, USA) on an ABI Prism 7900HT sequence detection system (Applied Biosystems, Foster City, CA, USA). *MTDH* primers were purchased from Invitrogen Life Technologies, the sequences were as follows: sense, 5′-CCATGATGGAAAGGAAGTTG-3′, antisense 5′-GAACCAACAGGAAATGATGC-3′ (189 bp); and β-actin sense, 5′-CATTAAGGAGAAGCTGTGCT-3′, and antisense 5′-GTTGAAGGTAGTTTCGTGGA-3′ (208 bp). The RT-qPCR amplification conditions were: 95°C for 5 min, 40 cycles at 94°C for 10 sec, 61°C for 20 sec and 72°C for 20 sec, followed by a final extension step at 72°C for 5 mins. The qPCR experiments were repeated at least three times. All samples were normalized to internal controls. The fold change in expression was then determined using the ΔΔCT method (19).

### Statistical analysis

SPSS 16.0 software (SPSS, Inc., Chicago, IL, USA) was used for statistical analysis. All data are expressed as the mean ± standard deviation of three independent experiments. A two-tailed Student’s t-test was used for comparisons between two independent groups. P<0.05 was considered to indicate a statistically significant difference.

## Results

### MTDH *is differentially expressed in the PC3, DU145 and LNCap cell lines*

*MTDH* expression was evaluated in the PC3, DU145 and LNCap cell lines using RT-qPCR and western blot analyses. Among the three cell lines, the relative expression levels of *MTDH* mRNA and protein in the DU145 (4.3±0.12; 0.72±0.04) and LNCap (4.13±0.03; 0.73±0.035) cells were significantly higher, as compared with those in the PC3 cells (0.97±0.08; 0.35±0.026) (P<0.01). However, no significant difference was observed between the DU145 and LNCap cells (P>0.05).

### Optimum concentration of cisplatin

**Figure 1:**
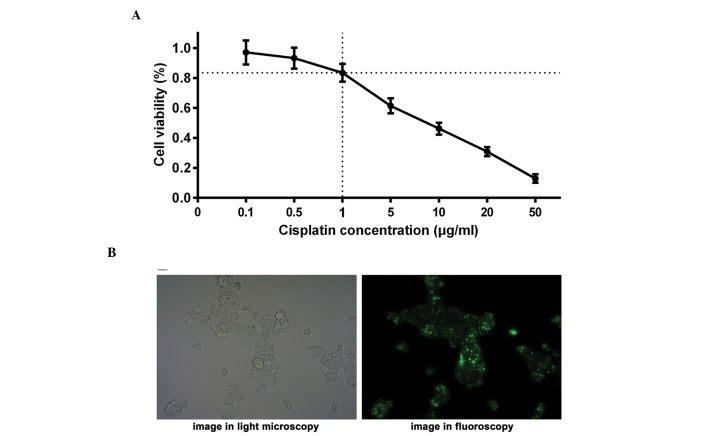
Determination of the optimum concentration of cisplatin and the transfection efficiency of the *MTDH* intervention sequence. (A) A curve was drawn to elucidate the optimum concentration of cisplatin, with cell viability on the y-axis and cisplatin concentration (0–50.0 *μ*g/ml) on the x-axis, indicating that treatment with 1.0 *μ*g/ml cisplatin for 24 h was appropriate for the assessment of prostate cancer cell chemotherapy sensitivity. (B) Compared with image in light microscopy, the cells transfected with *MTDH* siRNA present green fluorescent protein in image in fluoroscopy. The results indicate that >80% of cells were observed to be transfected, suggesting successful transfection. MTDH, metadherin; siRNA, small interfering RNA.

The LNCap cell line was treated with various concentrations of cisplatin (0, 0.1, 0.5, 1.0, 5.0, 10.0, 20.0 and 50.0 *μ*g/ml). An MTT assay was performed and a curve was constructed to identify the optimum concentration of cisplatin, with cell viability on the y-axis and cisplatin concentration on the x-axis (Fig. 1A). To avoid experimental errors due to excessive cell death induced by cisplatin, an appropriate concentration of cisplatin was selected to ensure that the viability of the LNCap cells remained at ~80% following treatment with cisplatin for 24 h. The results suggested that the cell viability of the LNCap cells was ~83% when treated with 1.0 *μ*g/ml cisplatin for 24 h, demonstrating that 1.0 *μ*g/ml cisplatin was the optimal concentration for further investigation. The half maximal inhibitory concentration (IC_50_) of cisplatin was also measured, indicating an IC_50_ of 7.1 *μ*g/ml in the LNCap cell line.

### MTDH intervention sequence effectively inhibits MTDH expression

**Figure 2:**
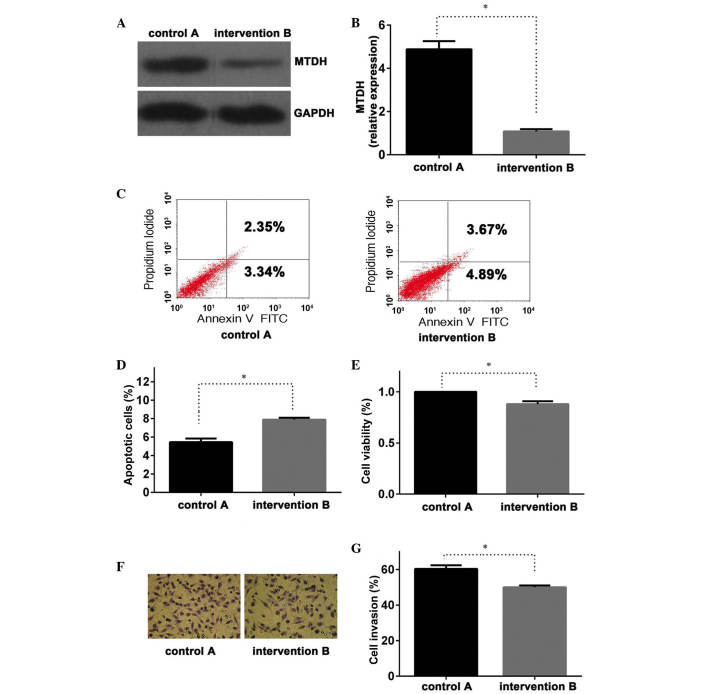
Suppression of *MTDH* expression promotes cell apoptosis and reduces the cell viability and invasive ability of prostate cancer cells. (A and B) Compared with the control group A, *MTDH* messenger RNA and protein expression were significantly lower in intervention group B (^*^P<0.01, compared with control group A). (C and D) An apoptosis assay, (E) an MTT assay for cell viability and (F and G) a Transwell chamber invasion assay with hematoxylin staining (magnification, x400) were also performed. The results suggested that intervention group B had a higher apoptotic rate, lower cell viability and weaker cell invasive ability than the control group A (^*^P<0.05, compared with control group A). *MTDH*, metadherin; FITC, fluorescein isothiocyanate.

**Figure 3:**
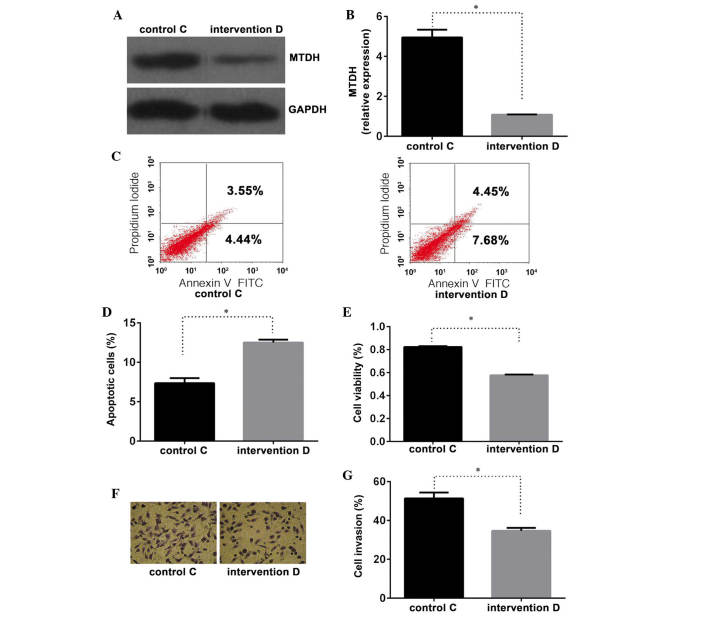
Repression of *MTDH* expression enhances prostate cancer cell sensitivity to cisplatin. (A and B) Compared with control group C, *MTDH* messenger RNA and protein expression were significantly lower in the intervention group D (^*^P<0.01). (C and D) An apoptosis assay, (E) an MTT assay for cell viability and (F and G) a Transwell chamber invasion assay with hematoxylin staining (magnification, x400) were also performed. The results suggested that intervention group D had a higher apoptotic rate, lower cell viability and weaker cell invasive ability than the control group C (^*^P<0.05). *MTDH*, metadherin; FITC, fluorescein isothiocyanate.

Prior to conduction of the present study, LNCap cells were divided into five groups: Two control groups Two control groups of conventional cultured LNCap cells and LNCap cells transfected with an empty vector, and three interventional groups of LNCap cells transfected with *MTDH*-744, *MTDH*-1432 and *MTDH*-1883. After a 48 h culture, the transfection efficiency of the *MTDH* intervention sequences in LNCap cells were assessed. It was observed that >80% of cells were transfected as elucidated via light microscopy and fluoroscopy (Fig. 1B), indicating that the transfection was successful and the subsequent experiments could be performed. In addition, *MTDH* protein expression levels were determined in each group, and the results indicated that *MTDH* protein expression levels were lowest in the LNCap cells transfected with *MTDH*-1432 (0.07±0.01), as compared with the other groups (P<0.01; conventional cultured LNCap 0.58±0.04, LNCap transfected with empty vector 0.55±0.04, LNCap transfected with *MTDH*-744 0.40±0.05 and with *MTDH*-1883 0.27±0.03). Therefore, *MTDH*-1432 was selected to perform further studies. The expression levels of *MTDH* mRNA and *MTDH* protein were significantly lower in group B compared with those of group A (P<0.01; Fig. 2A and B), with similar results observed in groups C and D (Fig. 3A and B), indicating that the *MTDH* intervention sequence was able to effectively inhibit *MTDH* expression.

### *Suppression of* MTDH *expression promotes cell apoptosis, reducing cell viability and invasion of PC*

The present study demonstrated an effective transfection of the *MTDH* intervention sequence (Figs. 1B, 2A and 2B). Once the LNCap cell line had been treated for 24 h, an apoptosis assay was performed. The results of the assay suggested that group B had a higher apoptotic rate than that of control group A (P<0.01; Fig. 2C and D), combined with the above results indicating that, compared with group A, *MTDH* mRNA and protein expression were significantly lower in group B, these findings may indicate that the repression of *MTDH* expression promoted PC cell apoptosis. Similar results were also observed in the MTT assay for cell viability (Fig. 2E) and Transwell chamber invasion assay, between groups A and B (Fig. 2F and G; P<0.05 and P<0.01, respectively). These results suggested that the inhibition of *MTDH* expression in group B may lead to a reduction in LNCap cell viability and invasive potential.

### Suppression of MTDH expression enhances PC cell sensitivity to cisplatin

Following the evaluation of various concentrations of cisplatin, cisplatin was administered at 1.0 *μ*g/ml for 24 h in order to assess PC cell sensitivity to cisplatin (Fig. 1A). Compared with control group C, cells transfected with the *MTDH* intervention sequence as well as cisplatin (group D) exhibited a higher apoptotic rate (Fig. 3C and D), lower cell viability (Fig. 3E) and decreased cellular invasiveness (Fig. 3F and G). These differences were confirmed to be statistically significant (P<0.01). Significant differences were identified in the *MTDH* mRNA and protein expression levels between the two groups (Fig. 3A and B), supporting the hypothesis that the repression of *MTDH* expression may enhance PC cell sensitivity to cisplatin.

### Suppression of MTDH expression inhibits the phosphoinositide 3-kinase (PI3K)/Akt signal transduction pathway

**Figure 4:**
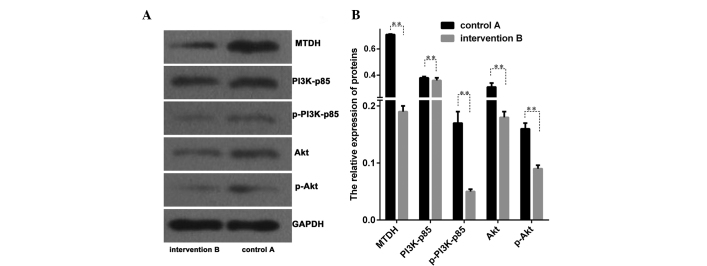
Levels of PI3K/Akt signal transduction pathway proteins. (A) Levels of *MTDH* associated proteins in the PI3K/Akt signal transduction pathway were evaluated in the control group A and the intervention group B by western blot analysis. (B) Quantification of western blot analysis. Expression levels of PI3K, p-PI3K, Akt and p-Akt were detected, and significant differences were identified between the two groups (^**^P<0.01). PI3K, phosphoinositide 3-kinase; p-AKT, phosphorylated Akt; p-PI3K, phosphorylated PI3K; *MTDH*, metadherin.

The protein levels associated with the PI3K/Akt signal transduction pathway of *MTDH* between groups A and B were evaluated. Proteins of PI3K, phosphorylated PI3K (p-PI3K), Akt and phosphorylated Akt (p-Akt) were analyzed and significant differences were identified between these two groups (P<0.01; Fig. 4A and B). The present results demonstrated that the PI3K/Akt signal transduction pathway may be inhibited following the transfection of LNCap cells with the *MTDH* intervention sequence.

## Discussion

Previous studies have revealed that *MTDH* functions as an oncogene (5–13). Overexpression of *MTDH* has been observed in multiple types of cancer, including breast cancer (3,8), hepatocellular carcinoma (5), human glioma (20), neuroblastoma (7,21), esophageal cancer (22), non-small-cell lung cancer (23), cervical cancer (24) and PC (11–12). In the present study, three common PC cell lines LNCap, DU145 and PC3 were used and the level of *MTDH* expression was detected using RT-qPCR and western blot analyses. Varying levels of expression were observed in the three cell lines, and it was observed that *MTDH* expression in the LNCap and DU145 cell lines was higher than that in the PC3 cell line. These results contradicted a previous study, in which *MTDH* expression in the DU145 and PC3 cell lines was observed to be markedly higher than that in the LNCap cell line (11). This may be due to differences in experimental conditions. It may be useful to adjust for these differences in future studies. The LNCap cell line was selected for the experiment as *MTDH* was detected at a relatively high expression level. The results of microscopy and fluoroscopy analyses suggested that transfection of the *MTDH* intervention sequence had occurred successfully. When comparing the experimental group subjected to transfection with the *MTDH* intervention sequence (group B) with the control group (group A), the repression of *MTDH* expression was observed to promote cell apoptosis, reduce cell viability and reduce the invasive potential of PC cells. These results were in agreement with those of previous studies (11,12).

*MTDH* has been observed to be important in conferring drug resistance in cancer treatment. Previous studies have demonstrated that knockdown of the *MTDH* gene led to an increase in breast cancer cell sensitivity to paclitaxel, doxorubicin and cisplatin (25,26). In hepatocellular carcinoma cells, *MTDH* is able to induce late SV40 factor leading to fluorouracil resistance as well as inducing the expression of multidrug resistance gene 1 and resulting in the development of doxorubicin resistance (15,26). In the present study, the inhibition of *MTDH* expression reduced tumor drug resistance in PC cells. The optimum concentration of cisplatin for use in the present experiments was evaluated and the administration of 1.0 *μ*g/ml cisplatin for 24 h was considered appropriate. The results indicated that the cisplatin-treated intervention group D exhibited a higher apoptotic rate, lower cell viability and decreased cellular invasiveness. All differences were significant compared with control group C.

As a classic signal transduction pathway, excessive activation of PI3K/Akt is closely associated with tumor development (27,28). A previous study observed that the *MTDH* gene is able to regulate the PI3K/Akt signal transduction pathway (29). The study demonstrated that *MTDH* gene activation may be suppressed by the PI3K/Akt inhibitors LY294002 and PTEN and in addition, the anti-apoptotic ability of *MTDH* may be reduced. In the present study, an examination was conducted in order to identify *MTDH*-mediated signaling pathways in PC cells. Following transfection with an *MTDH* interference fragment, the expression of proteins p-PI3K-p85 and p-Akt were significantly reduced compared with that of the control group. It was demonstrated that *MTDH* expression, associated with the PI3K-Akt signaling pathway may be involved in the biological behavior of PC.

In conclusion, the results of the present study demonstrated that the inhibition of *MTDH* expression may reduce the carcinogenic behavior of PC cells and increase their sensitivity to cisplatin. In addition, *MTDH* associated PI3K-Akt signaling pathways may be involved in PC development.

## References

[1] Ferlay J, Shin HR, Bray F (2010). Estimates of worldwide burden of cancer in 2008: GLOBOCAN 2008. Int J Cancer.

[2] Gasparini G, Longo R, Torino F, Morabito A (2005). Therapy of breast cancer with molecular targeting agents. Ann Oncol.

[3] Brown DM, Ruoslahti E (2004). Metadherin, a cell surface protein in breast tumors that mediates lung metastasis. Cancer Cell.

[4] Su ZZ, Kang DC, Chen Y (2002). Identification and cloning of human astrocyte genes displaying elevated expression after infection with HIV-1 or exposure to HIV-1 envelope glycoprotein by rapid subtraction hybridization. RaSH Oncogene.

[5] Yoo BK, Emdad L, Su ZZ (2009). Astrocyte elevated gene-1 regulates hepatocellular carcinoma development and progression. J Clin Invest.

[6] Chen D, Yoo BK, Santhekadur PK (2011). Insulin-like growth factor-binding protein-7 functions as a potential tumor suppressor in hepatocellular carcinoma. Clin Cancer Res.

[7] Liu H, Song X, Liu C, Xie L, Wei L, Sun R (2009). Knockdown of astrocyte elevated gene-1 inhibits proliferation and enhancing chemo-sensitivity to cisplatin or doxorubicin in neuroblastoma cells. J Exp Clin Cancer Res.

[8] Li J, Zhang N, Song LB (2008). Astrocyte elevated gene-1 is a novel prognostic marker for breast cancer progression and overall patient survival. Clin Cancer Res.

[9] Chen W, Ke Z, Shi H, Yang S, Wang L (2010). Overexpression of AEG-1 in renal cell carcinoma and its correlation with tumor nuclear grade and progression. Neoplasma.

[10] Yaari S, Jacob-Hirsch J, Amariglio N, Haklai R, Rechavi G, Kloog Y (2005). Disruption of cooperation between Ras and MycN in human neuroblastoma cells promotes growth arrest. Clin Cancer Res.

[11] Kikuno N, Shiina H, Urakami S (2007). Knockdown of astrocyte-elevated gene-1 inhibits prostate cancer progression through upregulation of FOXO3a activity. Oncogene.

[12] Thirkettle HJ, Girling J, Warren AY (2009). LYRIC/AEG-1 is targeted to different subcellular compartments by ubiquiti-nylation and intrinsic nuclear localization signals. Clin Cancer Res.

[13] Ash SC, Yang DQ, Britt DE (2008). LYRIC/AEG-1 overexpression modulates BCCIPalpha protein levels in prostate tumor cells. Biochem Biophys Res Commun.

[14] Hu G, Chong RA, Yang Q (2009). MTDH activation by 8q22 genomic gain promotes chemoresistance and metastasis of poor-prognosis breast cancer. Cancer Cell.

[15] Yoo BK, Gredler R, Vozhilla N (2009). Identification of genes conferring resistance to 5-fluorouracil. Proc Natl Acad Sci USA.

[16] Desoize B, Madoulet C (2002). Particular aspects of platinum compounds used at present in cancer treatment. Crit Rev Oncol Hematol.

[17] Sciuto R, Festa A, Rea S (2002). Effects of low-dose cisplatin on 89Sr therapy for painful bone metastases from prostate cancer: A randomized clinical trial. J Nucl Med.

[18] Dhar S, Kolishetti N, Lippard SJ, Farokhzad OC (2011). Targeted delivery of a cisplatin prodrug for safer and more effective prostate cancer therapy in vivo. Proc Natl Acad Sci USA.

[19] Livak KJ, Schmittgen TD (2001). Analysis of relative gene expression data using real-time quantitative PCR and the 2(-Delta Delta C(T)) Method. Methods.

[20] Liu L, Wu J, Ying Z (2010). Astrocyte elevated gene-1 upregulates matrix metalloproteinase-9 and induces human glioma invasion. Cancer Res.

[21] Lee SG, Jeon HY, Su ZZ (2009). Astrocyte elevated gene-1 contributes to the pathogenesis of neuroblastoma. Oncogene.

[22] Yu C, Chen K, Zheng H (2009). Overexpression of astrocyte elevated gene-1 (AEG-1) is associated with esophageal squamous cell carcinoma (ESCC) progression and pathogenesis. Carcinogenesis.

[23] Song L, Li W, Zhang H (2009). Over-expression of AEG-1 significantly associates with tumour aggressiveness and poor prognosis in human non-small cell lung cancer. J Pathol.

[24] Emdad L, Sarkar D, Su ZZ (2006). Activation of the nuclear factor kappaB pathway by astrocyte elevated gene-1: implications for tumor progression and metastasis. Cancer Res.

[25] Harris T, Jimenez L, Kawachi N (2012). Low-level expression of miR-375 correlates with poor outcome and metastasis while altering the invasive properties of head and neck squamous cell carcinomas. Am J Pathol.

[26] Yoo BK, Chen D, Su ZZ (2010). Molecular mechanism of chemoresistance by astrocyte elevated gene-1. Cancer Res.

[27] Fresno VJ, Casado E, de Castro J, Cejas P, Belda-Iniesta C, González-Barón M (2004). PI3K/Akt signalling pathway and cancer. Cancer Treat Rev.

[28] Osaki M, Oshimura M, Ito H (2004). PI3K-Akt pathway: its functions and alterations in human cancer. Apoptosis.

[29] Lee SG, Su ZZ, Emdad L, Sarkar D, Franke TF, Fisher PB (2008). Astrocyte elevated gene-1 activates cell survival pathways through PI3K-Akt signaling. Oncogene.

